# Efficacy and safety of fecal microbiota transplantation for chronic insomnia in adults: a real world study

**DOI:** 10.3389/fmicb.2023.1299816

**Published:** 2023-11-21

**Authors:** Haiming Fang, Tingting Yao, Wanli Li, Na Pan, Hang Xu, Qian Zhao, Yuan Su, Kangwei Xiong, Jiajia Wang

**Affiliations:** ^1^Department of Gastroenterology, The Second Affiliated Hospital of Anhui Medical University, Hefei, Anhui, China; ^2^Center for Gut Microbiota Diagnosis and Treatment, The Second Affiliated Hospital of Anhui Medical University, Hefei, Anhui, China; ^3^Department of Pharmacology, School of Basic Medical Sciences of Anhui Medical University, Hefei, Anhui, China; ^4^School of Medicine, Anhui University of Science and Technology, Huainan, Anhui, China

**Keywords:** chronic insomnia, brain-gut axis, fecal microbiota transplantation, gut microbiota, dysbiosis

## Abstract

**Objective:**

To assess the efficacy and safety of fecal microbiota transplantation (FMT) for adult chronic insomnia.

**Methods:**

Patients treated with FMT for chronic diseases were divided into chronic insomnia and non-insomnia group. The primary endpoint was the efficacy of FMT for insomnia 4 weeks after treatment, the secondary endpoints included the impacts of FMT on anxiety, depression, health-related quality of life, gut microbiota, and adverse events associated with FMT. Insomnia Severity Index (ISI) and Pittsburgh Sleep Quality Index (PSQI) were utilized to assess the efficacy of FMT on insomnia, self-rating anxiety/depression scale [Zung Self-Rating Anxiety Scale (SAS), Zung Self-Rating Depression Scale (SDS)] was employed to evaluate anxiety and depression. Quality of life was evaluated by SF-36. 16S rRNA sequencing was employed to analyze the gut microbiota and correlation analysis was performed.

**Results:**

Forty patients met the inclusion criteria and seven were excluded. 33 patients were enrolled and stratified into chronic insomnia group (*N* = 17) and non-insomnia group (*N* = 16). Compared to baseline, FMT significantly ameliorated the ISI (17.31 ± 5.12 vs. 5.38 ± 5.99), PSQI (14.56 ± 2.13 vs. 6.63 ± 4.67), SAS (54.25 ± 8.90 vs. 43.68 ± 10.64) and SDS (57.43 ± 10.96 vs. 50.68 ± 15.27) score and quality of life of chronic insomnia patients. 76.47% (13/17) of insomnia patients achieved the primary endpoints. In chronic insomnia patients, the relative abundance of *Eggerthella* marked enhanced at baseline, while the relative abundance of *Lactobacillus, Bifidobacterium, Turicibacter, Anaerostipes*, and *Eisenbergiella* significantly increased after FMT treatment, the latter positive correlated with the efficacy of FMT. Encouragingly, FMT also improved the sleep quality of non-insomnia patients.

**Conclusion:**

*Eggerthella* may potentially serve as a distinctive genus associated with chronic insomnia. FMT maybe a novel treatment option for adults with chronic insomnia and provide an alternative to traditional treatments for insomnia. The effects were positive correlated with the augmentation of probiotics, such as *Bifidobacterium*, *Lactobacillus*, *Turicibacter*, and *Fusobacterium*.

## Introduction

The physiological imperative of sleep for all living organisms is emphasized by the fact that adult humans spend average around one-third of their lives engaged in this essential activity. However, sleep disorders have emerged as a prevalent global public health concern in modern society (Han et al., 2022). The term insomnia refers to the unwelcome experience of difficulty in initiating or maintaining sleep, awakening during the night with difficulty returning to sleep, or early morning awakenings, resulting in impaired daytime functioning (Zhang et al., 2022). The prevalence of insomnia is considerably high, ranging from 6.6 to 12% in the general population, with its occurrence reported by 19 to 50% of adults (Koopman et al., 2020; Dos Reis et al., 2021). Nearly 50% of the elderly population suffer from chronic insomnia, which is characterized by fragmented sleep and early morning awakening (Reid et al., 2010; Ryden et al., 2019). The prevalence of this condition is higher in women than men, with a ratio ranging from 1.3 to 1.7, and it may manifest or worsen during hormonal fluctuations such as premenstrual, peripartum, and postmenopausal periods (Suh et al., 2018). Up to 40% of insomnia sufferers also experience comorbid psychiatric disorders, with depression and anxiety being the most prevalent (Roth, 2007). The occurrence of insomnia can be acute, intermittent, or chronic, making it one of the most prevalent forms of sleep disturbance. Insomnia is closely linked to the etiology and progression of a range of disorders, such as obesity, type II diabetes, and cardiovascular diseases (Liu et al., 2019).

The objective of treating insomnia is to enhance the patient’s sleep experience and quality of life while minimizing any potential adverse effects. The current guidelines advocate a combination of pharmacological and non-pharmacological interventions, with cognitive behavioral therapy for insomnia (CBTI) being the preferred approach due to its proven efficacy, safety, and enduring benefits. Pharmacotherapy can be considered as an alternative in cases where CBTI fails to yield satisfactory outcomes (Sutton, 2021). Compared to no treatment or sleep hygiene advice, CBTI demonstrated superior efficacy in improving nocturnal sleep measures among adults with and without psychiatric and/or medical comorbidities, as well as those taking concomitant sleep medication (van Straten et al., 2018). However, each of these treatment modalities has limitations in clinical practice. While population surveys inquire about insomnia symptoms, sleep medicine trials and guidelines on insomnia treatment solely focus on individuals who meet the criteria for insomnia disorder. Therefore, these guidelines do not apply universally to all individuals experiencing symptoms of insomnia. Both CBTI and pharmacologic therapy yield only modest effects on objective sleep measures. The utilization of CBTI is further associated with increased access barriers in comparison to pharmacological treatments, which are commonly employed for the management of insomnia symptoms (Sutton, 2021). Hence, this underscores the imperative to develop innovative (non-pharmacological) treatment modalities for the management of chronic insomnia in adult individuals.

With the increasing comprehension of brain-gut axis, it is becoming increasingly clear that the relationship between host and microbiota is a key area of impact for populations (Cryan et al., 2020). It has been hypothesized that the gut microbiota may exert a significant impact on neurological disorders throughout the lifespan, encompassing early-onset conditions such as autism and attention deficit hyperactivity disorder (ADHD), adult-onset diseases like multiple sclerosis (MS) and major depressive disorder (MDD), as well as age-related afflictions such as Alzheimer’s disease (AD) and Parkinson’s disease (PD) (Cox et al., 2019). The classification of psychiatric disorders is based on diagnostic categories encompassing a wide range of symptoms; however, patients with the same diagnosis often exhibit significant clinical. This clinical heterogeneity, coupled with the incomplete knowledge of the biological mechanisms of psychiatric disorders and their comorbidity with other conditions, have so far contributed towards the limited efficacy of current treatments. Multi-omics technologies (e.g., genomics, transcriptomics, proteomics, and lipidomics) provide a promising avenue for the identification of biomarkers in psychiatry, enabling patient stratification within a diagnosis, facilitating more targeted treatment options, and predicting treatment response (Sathyanarayanan et al., 2023). Furthermore, omic technologies also play pivotal roles in bacterial resistance reserach (Bongiorno et al., 2023) The gut microbiota has emerged as a promising therapeutic target for psychiatric disorders such as depression, Parkinson’s disease, and dementia (Ho et al., 2021). Recently, a range of probiotics have been reported to possess effective stress-modulating and anxiolytic effects on stressed individuals by maintaining intestinal homeostasis, enhancing mucosal and systemic immunity, and regulating gut microbiota metabolism. The findings suggest that probiotics show potential as psychobiotics for mitigating psychiatric disorders, and they offer a promising non-pharmacological therapy for insomnia. For instance, *Lactobacillus*, a prominent bacterium in the gut microbiota, possesses the potential to modulate the central nervous system through the vagus nerve and exert an impact on sleep patterns. The utilization of probiotics for sleep and emotional disorders, such as insomnia, stress, anxiety, and depression, is gaining significant momentum (Irwin et al., 2020; Tian et al., 2022).

Fecal microbiota transplantation (FMT) commonly refers to the transfer of gut microbiota from healthy donors to patients with dysbiosis-related diseases, aiming to restore homeostasis of the gut microbiota and achieve treatment goals (Fang et al., 2021). FMT has demonstrated remarkable efficacy in treating recurrent *Clostridium difficile* infection, surpassing that of vancomycin in randomized controlled trials and exhibiting promising results for managing inflammatory bowel disease (IBD) in both pediatric and adult populations (Fang et al., 2018). Moreover, gut microbiota is involved in several mechanisms sustaining the model of the “gut-liver axis.” Evaluation of changes in the gut microbiota composition in liver transplantation patients is essential for monitoring transplant success and potentially implement appropriate preventive measures (Abenavoli et al., 2023). FMT has shown preclinical and initial clinical promising results in non-alcoholic fatty liver disease treatment through re-modulation of microbial dysbiosis (Abenavoli et al., 2022). This highlights FMT as a potential therapeutic option with great promise (Fang et al., 2018). Germ-free mice receiving FMT from participants who underwent overnight sleep deprivation exhibited an increase in inflammatory states and enhanced intestinal barrier permeability, similar to that observed in specific pathogen-free (SPF) mice after sleep deprivation. These findings suggest that gut dysbiosis contributes to both peripheral and central inflammatory processes induced by sleep loss, highlighting the potential of manipulating the microbiota as a therapeutic intervention for mitigating the detrimental consequences of sleep deprivation (Wang et al., 2021). A pilot study revealed that FMT had a beneficial impact on sleep quality as well as patients’ mood of FMT, regardless of gastrointestinal symptom change in patients with irritable bowel syndrome (IBS), functional diarrhea (FD) or functional constipation (Kurokawa et al., 2018). However, it is important to note that the scope of above study was limited to investigating the potential enhancement of sleep quality specifically in patients with aforementioned intestinal diseases. Furthermore, the assessment relied solely on sleep-related sub-scores derived from the Hamilton Rating Scale for Depression (HAM-D). FMT could improve sleep quality among IBS patients experiencing poor sleep (Zhang Z. et al., 2023). However, to the best of our knowledge, there is a paucity of studies specifically investigating the efficacy and safety of FMT in individuals afflicted with chronic insomnia. The aim of our study was to investigate whether FMT could potentially ameliorate self-reported sleep quality, mood disturbances, daytime dysfunction and health-related quality of life in adults with chronic insomnia.

## Methods

### Patients and study design

The present study is an observational study conducted in a real-world setting, serving as a sub-analysis of an ongoing investigation into the efficacy and safety of FMT for refractory chronic diseases in adult patients. These disorders encompass recurrent *Clostridium difficile* infection (rCDI), irritable bowel syndrome (IBS), chronic functional constipation, ulcerative colitis (UC), or Parkinson’s disease with constipation. The study employed a parallel pre-post design to investigate the impact of FMT on self-reported sleep quality, mood, quality of life, and gut microbiota composition at baseline and post-intervention (4 weeks after FMT). The study was conducted at the Department of Gastroenterology, the Second Affiliated Hospital of Anhui Medical University. Participants were enrolled from September 2020 to July 2022.

According to the Diagnostic and Statistical Manual of Mental Disorders, 5th Edition (DSM-5), the diagnostic criteria for insomnia necessitate the presence of at least one of the following symptoms: difficulty initiating sleep, difficulty maintaining sleep, or early morning awakening with an inability to return to sleep. Chronic insomnia is defined as experiencing any of these symptoms for a minimum of three nights per week over a period lasting 3 months or longer (Seow et al., 2018). The sleep quality was assessed using the Insomnia Severity Index (ISI) and Pittsburgh Sleep Quality Index (PSQI), which can be considered as objective assessment methods for subjective experiences (Besedovsky et al., 2019). The present study employed the ISI as a screening tool to identify clinical insomnia, whereby participants were classified into either the chronic insomnia group or control group based on their PSQI scores. Individuals who achieved a PSQI score of 11 or higher and had not undergone CBTI within the preceding 4 weeks were included in the chronic insomnia group, whereas those with a score below 11 were assigned to the control group representing typical sleep patterns. Patients with chronic insomnia who were pregnant, had a history of FMT treatment, had been exposed to probiotics or prebiotics within the past 4 weeks, exhibited evidence of infection such as cytomegalovirus or Epstein–Barr virus, or required parenteral antibiotics were excluded. The exclusion criteria also encompassed patients with comorbidities such as cardiovascular disease, pulmonary disease, cerebrovascular disease, a history of gastrointestinal malignancy or polyps, and those who had undergone abdominal surgery. Participants who were ineligible for endoscopy or had contraindications were also excluded from the study. The Ethics Committee of the Second Affiliated Hospital of Anhui Medical University granted approval for this study [No. YX2019-039(F2)] and [No. YX2019-040(F2)], and Trial registration: ChiCTR, ChiCTR2000030080 and ChiCTR1900027238. All patients provided written informed consent.

### Donor-recipient matching model for donor selection and preparation of donor fecal microbiota solutions

Potential healthy stool donors were identified through a rigorous screening questionnaire, followed by a comprehensive medical interview and examination, as well as blood and stool testing to minimize the risk of disease transmission, in accordance with previously established protocols in our center (Fang et al., 2021). Bioinformatics analyzes were conducted, and the composition and stability of gut microbiota in stool samples obtained from eligible donors were dynamically monitored using metagenomics. Six representative microbial characteristics, namely richness, distance, beneficial taxa, harmful taxa, beneficial pathways and harmful pathways were employed as indicators for constructing a donor-recipient matching model using the Analytic Hierarchy Process (AHP) (Zhang B. et al., 2023). A minimum of 100 grams of fresh stool sample was required for each donation. The fresh stool (25%) should be homogenized with normal saline (60%) and pharmaceutical grade glycerol (15%), and then processed in an automatic stirring and separation machine. The specifications, appearance, quantity, and weight of all products were thoroughly examined before being immediately frozen at −80°C. Based on the donor-recipient matching model, a total of thirteen healthy donors were included, with an average age of 22.08 ± 2.29 (19–25) years old, comprising eight males and five females. The mean BMI was documented as 21.62 ± 1.36 (18.9–23.5) kg/m^2^, while the health assessment indicated normal findings with no history of smoking or alcohol consumption and absence of oral or gut inflammation.

### FMT procedure

The participants were given explicit instructions to abstain from consuming any other probiotic products or antibiotics throughout the entire duration of the study, and a light diet was recommended for 2–3 days prior to FMT. The participants received bowel preparation with polyethylene glycol electrolyte dissolved in 2 L of water, which is an isotonic lavage fluid for the entire intestine. This lavage fluid contains 125 mmol/L sodium ions, 10 mmol/L potassium ions, 20 mmol/L bicarbonate ions, 40 mmol/L sulfate ions, and 35 mmol/L chloride ions. The gastroscopy and colonoscopy procedures were conducted under propofol sedation, while the nasoduodenal graft tube was inserted below the descending portion of the duodenum 4–6 h prior to FMT. The participants underwent FMT treatments administered via the nasoduodenal route. Specifically, a total of 600 mL of donor fecal slurry was introduced into the gastrointestinal tract through a naso-duodenal tube for three consecutive days (200 mL/d × 3d). Following completion of transplantation, all recipients were instructed to maintain a semi-recumbent position for at least 60 min without defecation. Stool specimens were collected at baseline (prior to FMT) and post-FMT (at least 4 weeks after treatment).

### Assessments of the primary and secondary endpoints

The primary endpoints of this study were to evaluate the efficacy of FMT in improving sleep quality among patients with chronic insomnia, 4 weeks post-FMT. The secondary endpoints encompassed the evaluation of the impact of FMT on anxiety and depression, health-related quality of life, as well as any adverse events associated with FMT, which were documented throughout both the treatment and follow-up periods. Questionnaires were administered at baseline and post-treatment (4 weeks after FMT). The ISI and PSQI were employed to assess subjective sleep quality and severity of insomnia. The PSQI, a 19-item measure, was utilized to evaluate sleep quality and disturbances over a one-month period. From this assessment, seven sub-scores were derived: sleep quality, sleep latency, sleep duration, habitual sleep efficiency, sleep disturbances, use of sleeping medication, and daytime dysfunction. Meanwhile, the Zung Self-Rating Anxiety Scale (SAS) and the Zung Self-Rating Depression Scale (SDS) were employed to evaluate participants’ mental health status. They both had 20 questions, each with a score from 0 to 4. The total scores of 20 items were used as raw scores, and the index scores were derived from the raw scores multiplied by a factor of 1.25. According to the Chinese population, the SAS standard score cut-off value was established at 50 points, with scores ranging from 50 to 59 indicating mild anxiety, scores between 60 and 69 denoting moderate anxiety, and scores of 70 or higher representing severe anxiety. The SDS standard score cut-off value was set at 53 points, with scores ranging from 53 to 62 indicating mild depression, scores between 63 and 72 signifying moderate depression, and scores of 73 or higher reflecting severe depression. The severity of depression was determined by calculating the cumulative score of each item divided by 80. A score below 0.5 indicates the absence of depression, while a score ranging from 0.5 to 0.59 suggests mild to moderate depression, a score between 0.6 and 0.69 indicates moderate to severe depression, and a score above 0.7 is indicative of major depression. Health-related quality of life was evaluated using the SF-36 questionnaire.

### Evaluation and analysis of the fecal microbiota through 16S rRNA sequencing

The donors provided fresh fecal samples, and patients’ pre- and post-FMT samples were collected using a sterile collection spoon. Subsequently, the samples were preserved in 3 mL of solution and stored at −80°C for subsequent analysis. The gut microbiota was analyzed using 16S rRNA sequencing. The V3-V4 hypervariable region of the 16S rRNA gene was amplified through high-throughput sequencing on the Illumina MiSeq platform, and the raw sequencing data from these stool samples were processed into operational taxonomic units at a similarity level of 97%, following previously established protocols (Fang et al., 2021).

### Statistical analyzes and visualization

A intention-to-treat analysis was conducted, wherein baseline demographic, medication, and disease parameters - including disease duration, severity, and extent - were presented using mean ± standard deviation ( $\bar{x}$ ±s) or frequencies (percentages). The Chi-square test was utilized to compare categorical variables between groups, while the Student’s t-test was employed to compare clinical response in both groups. The statistical analysis was conducted using SPSS Statistics v25.0, while the plotting was performed with GraphPad Prism 8. A significance level of *p* < 0.05 was considered.

The alpha diversity estimates were computed based on an evenly rarefied OTU abundance matrix, encompassing observed richness, species, and the Shannon, Simpson, ACE, and Chao1 indices using the R package vegan. Beta diversity was evaluated using the Bray-Curtis distance metric based on an evenly rarefied table of OTU abundances. LEfSe analysis was conducted to identify taxa exhibiting differential abundances across groups. Additionally, indicator analysis at the genus level was performed. To predict the functional content of metagenomics, phylogenetic investigation of communities by reconstruction of unobserved states (PICRUSt) was employed to forecast the presence of genes based on 16S data between groups. The metagenome predictions were conducted using the predicted metagenomes.py script. The significant difference analysis was performed using ANOVA. The results were visualized using a custom R script based on ggplot2, in accordance with previously reported protocols (Fang et al., 2021).

## Results

### Demographic characteristics of patients

**Figure 1:**
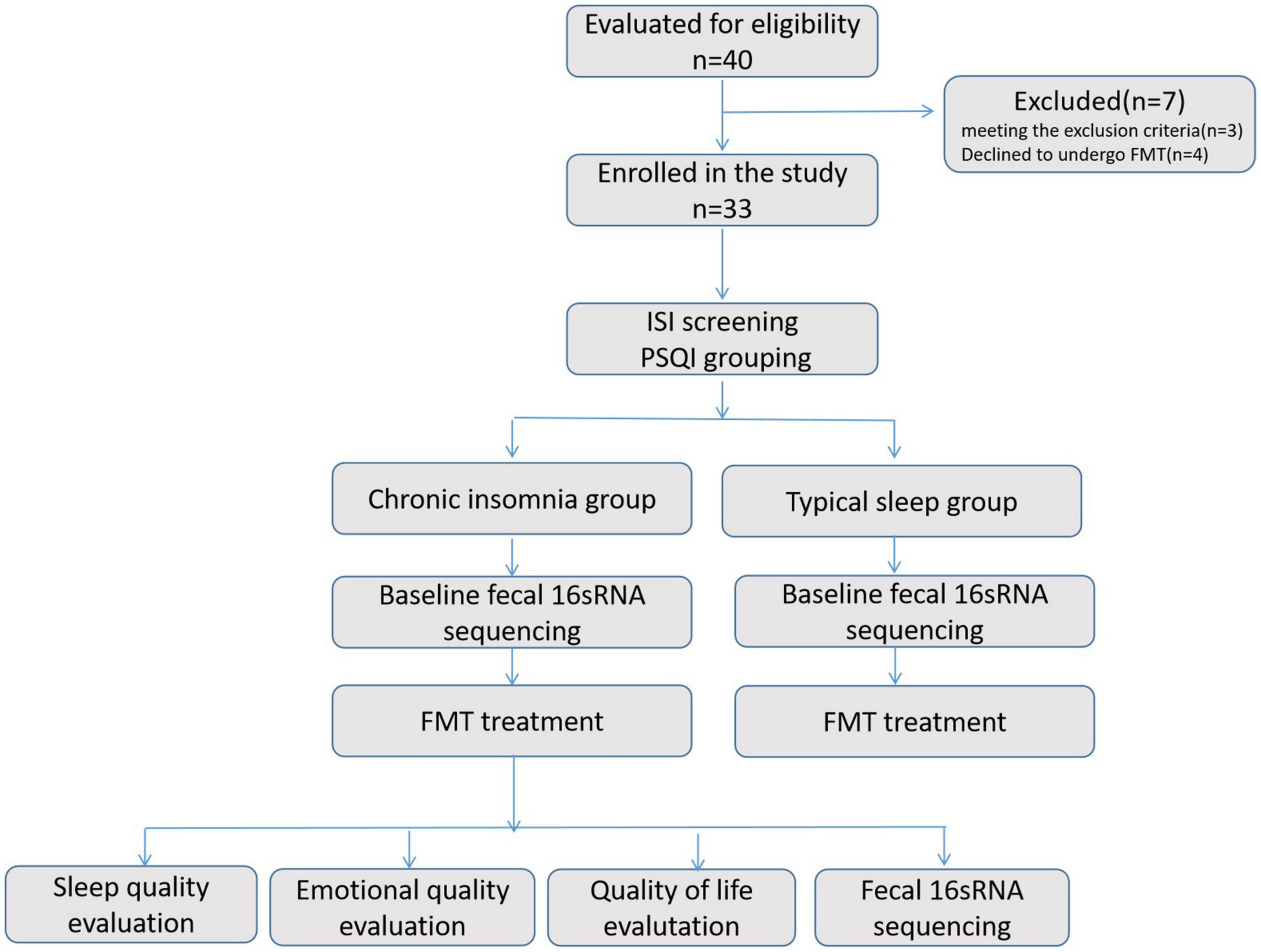
The study flow chart illustrates the process of recruitment and group assignment.

The present study was conducted in a real-world setting. Forty patients met the inclusion criteria from September 2020 to July 2022, seven patients were excluded, including 3 who met the exclusion criteria and 4 who refused FMT therapy. Consequently, a total of 33 patients were enrolled in this study, including 25 females and 8 males. The disease spectrum encompasses a range of conditions including rCDI, IBS, chronic constipation, UC, and Parkinson’s disease. The 33 patients were assigned to either the chronic insomnia group (*N* = 17) with an average age of 54.29 ± 19.76 years old or the typical sleep group (*N* = 16) with an average age of 56.81 ± 17.81 years old based on their PSQI scores. Interestingly, in this study, the prevalence rate of insomnia was found to be significantly higher among women compared to men, with a ratio of 15 to 2 (Table 1). There was no statistically significant disparity observed in terms of demographic characteristics and disease types between the two groups. The three consecutive days FMT treatment was administered to all 33 patients for their aforementioned diseases. The study flow chart illustrate the process of recruitment and group assignment (Figure 1), illustrating the baseline demographic features and clinical characteristics of patients with chronic insomnia.

**Table 1:** Demographic and clinical features of patient.

Accompanied symptoms	Chronic insomnia	Control group
Numbers	17	16
Female/Male	15/2	11/5
Age	54.29 ± 19.76	56.81 ± 17.81
Other diagnosis		
Chronic constipation	7	8
IBS	4	0
UC	1	2
Parkinson disease	2	2
rCDI	3	4
ISI score	17.31 ± 5.12	2.40 ± 2.47
PSQI score	14.56 ± 2.13	5.40 ± 2.58
FMT times	200 mL/d × 3d	200 mL/d × 3d
FMT route	Nasoduodenal	Nasoduodenal

### Effects of FMT on sleep quality in chronic insomnia patients

**Figure 2:**
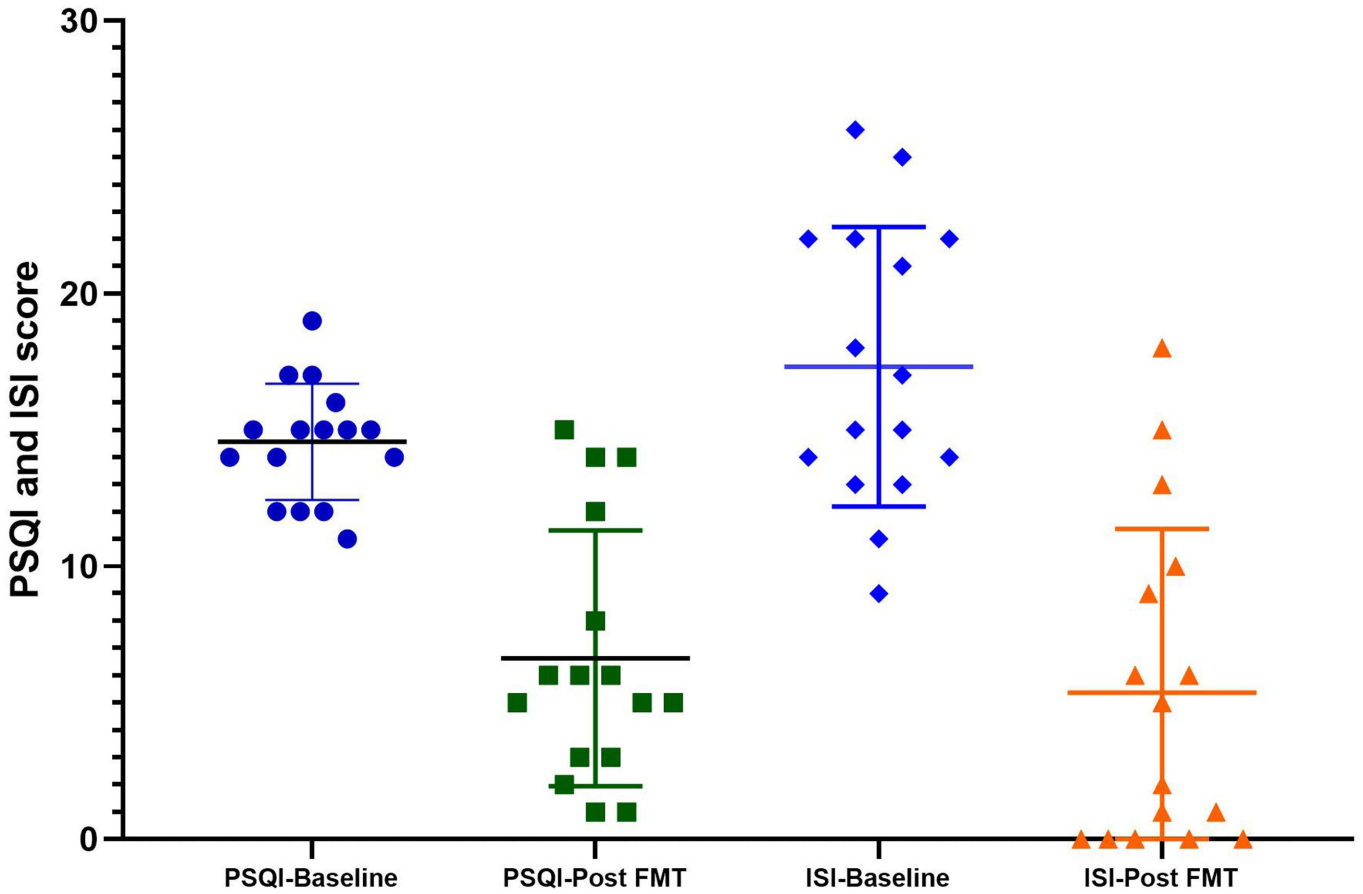
The PSQI and ISI scores at baseline and post FMT in the insomnia group. Compared to baseline, FMT significantly improved both PSQI (*p* = 0.0041) and ISI scores (*p* = 0.0027).

In the chronic insomnia group, the patients exhibited symptoms such as difficulty initiating sleep, early morning awakening, reduced total sleep time, and daytime fatigue. The initial ISI score was 17.31 ± 5.12, while the baseline PSQI score was 14.56 ± 2.13. Following FMT treatment, there was a significant reduction in the PSQI score during the four-week follow-up period, averaging at 6.63 ± 4.67. FMT demonstrated a noteworthy improvement in PSQI scores compared to the baseline value (*p* = 0.0041). Additionally, Compared to the baseline value, the ISI value also demonstrated a significant decrease with an average of 5.38 ± 5.99 (*p* = 0.0027, Figure 2). 76.47% (13/17) of insomnia patients achieved the primary endpoints after undergoing FMT treatment.

**Figure 3:**
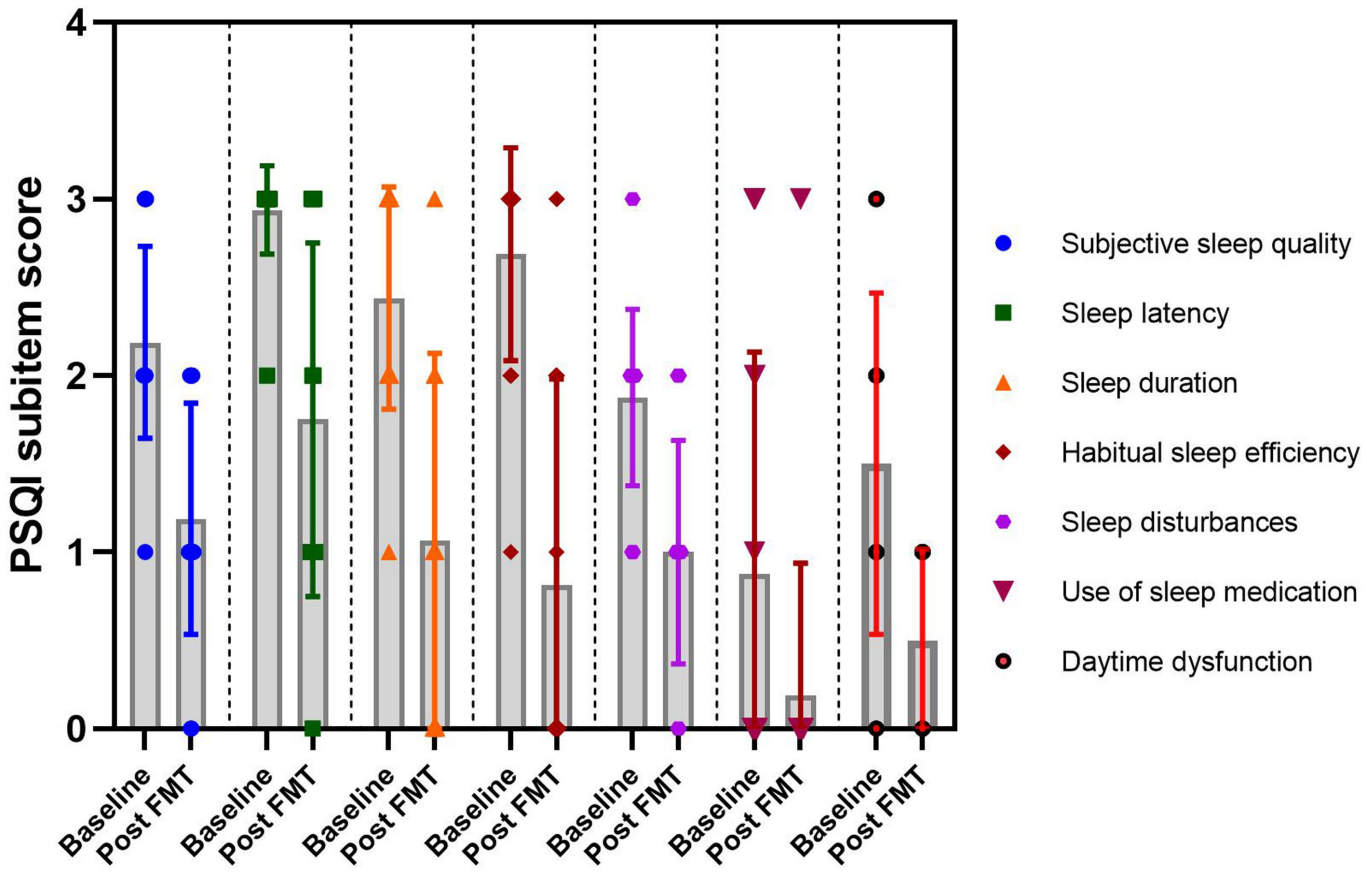
The PSQI sub-item score at baseline and post FMT in the insomnia group.

According to the PSQI score sub-item, FMT demonstrated significant improvements in insomnia by reducing sleep onset latency, enhancing sleep efficiency, and subsequently alleviating symptoms of daytime fatigue (Figure 3).

Encouragingly, patients without insomnia who underwent FMT also reported improved sleep quality with reduced sleep onset latencies, decreased wake time after sleep onset, and increased sleep efficiency (data not shown).

### Effects of FMT on anxiety and depression symptoms in chronic insomnia patients

**Figure 4:**
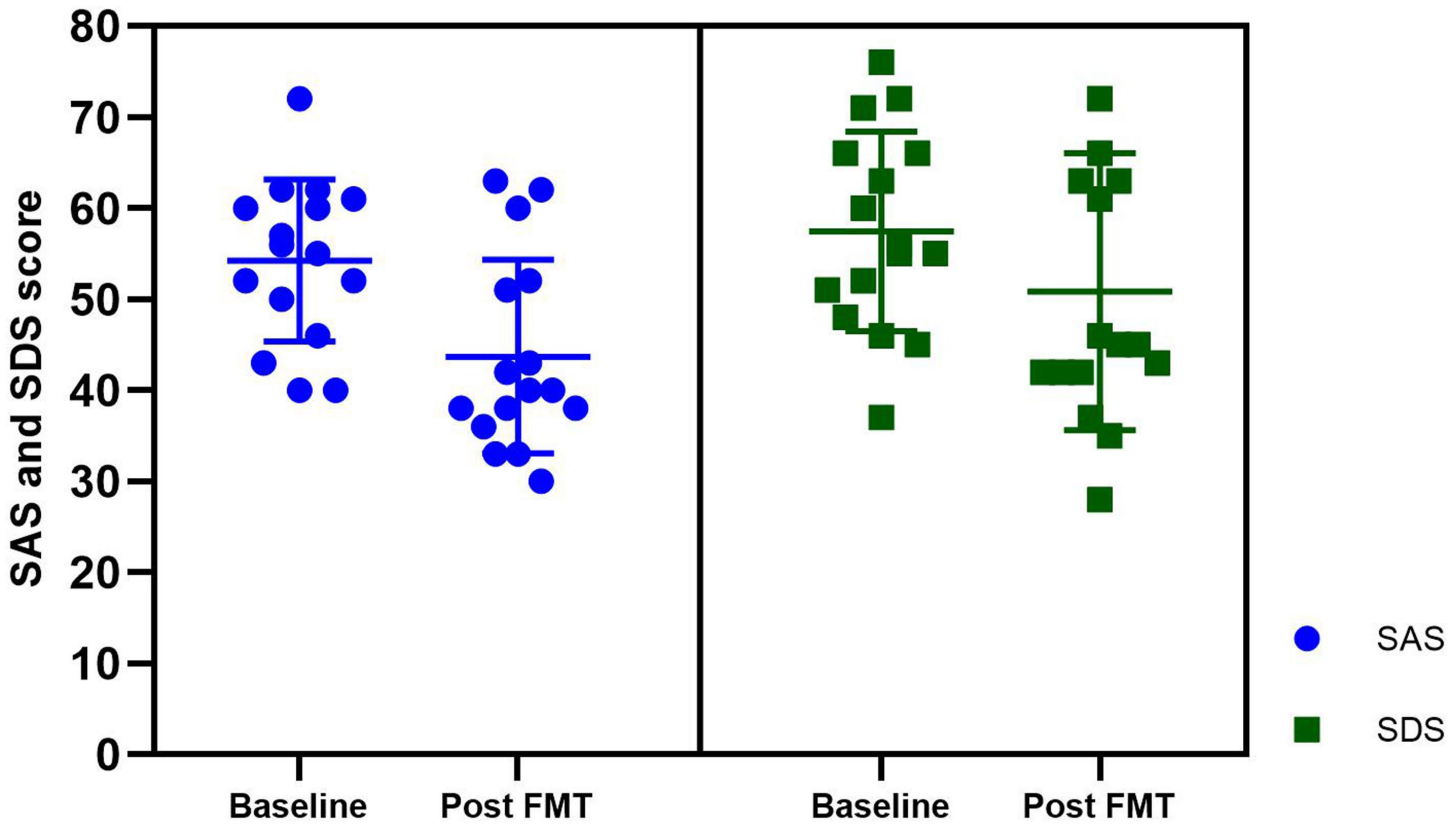
The anxiety and depression scores at baseline and post FMT in the insomnia group. SAS, Self-rating anxiety scale; SDS, Self-rating depression scale.

In this study, we observed varying degrees of anxiety and depression symptoms among patients with insomnia, The average baseline scores for the SAS and SDS in these patients were 54.25 ± 8.90 and 57.43 ± 10.96, respectively. The patients with comorbid chronic insomnia and chronic diseases displayed mild levels of anxiety and depression. After FMT treatment, the mean SAS score decreased to 43.68 ± 10.64 and the mean SDS score decreased to 50.68 ± 15.27. Following FMT treatment, there was a significant reduction in self-reported scores for anxiety and depression, indicating that FMT may effectively ameliorate psychiatric symptoms in patients with chronic diseases (Figure 4).

### Effects of FMT on quality of life in chronic insomnia patients

**Figure 5:**
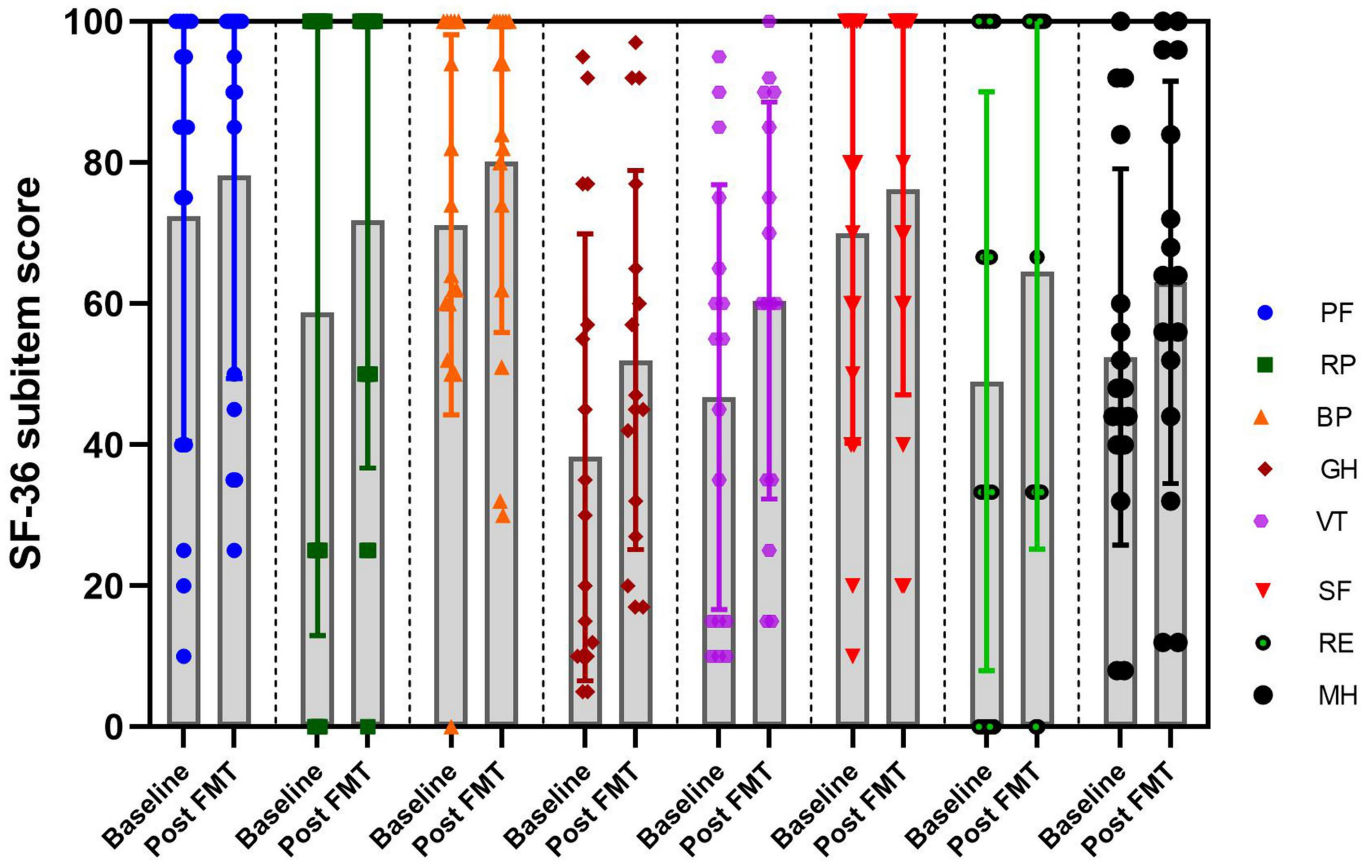
The plot of the SF-36 sub-item score at baseline and post FMT in the insomnia group. RF, Physical functioning; RP, Role-physical; BP, Body pain; GH, General health; VT, Vitality; SF, Social functioning; RE, Role-emotional; MH, Mental health.

The SF-36 is an 8-dimensional instrument specifically designed for the professional assessment of health-related quality of life (HRQoL). In order to investigate the impact of FMT on the quality of life in patients with chronic insomnia. We assessed the scores of the SF-36 questionnaire at baseline and 4 weeks after undergoing FMT treatment. As results, the FMT treatment demonstrated significant improvements in physical functioning, role-physical, body pain, general health, vitality, social functioning, role-emotional and mental health compared to the baseline (Figure 5), indicating FMT has the potential to enhance the quality of life for patients with chronic diseases.

### Safety of FMT in the treatment of chronic insomnia

Adverse events were recorded after FMT and follow-up. All patients could tolerate FMT well, and no serious adverse events occurred. No FMT-related serious adverse events were reported during the study period.

### Comparison of gut microbiota characteristics in patients with chronic diseases comorbid chronic insomnia or not

To investigate the potential variations in gut microbiota among individuals with chronic insomnia, the fecal microbiomes of the two groups at baseline were analyzed by sequencing the 16 s rRNA gene. Based on the observed plateau in the rarefaction curve of the current sequencing data, it can be inferred that a substantial portion of diversity has already been captured across all samples. Compared to the non-insomnia group, the insomnia group did not exhibit a significant difference in the alpha and beta diversity index. The linear discriminant analysis effect size (LEfSe) was subsequently employed to identify differential microbial communities between the two groups. However, no statistically significant differences were observed at the phylum, class, or family levels, potentially due to the similarity in disease spectrum between the two groups. Interestingly, at the order level, there was a notable increase in the relative abundance of fecal *Rhizobiales* in the non-insomnia group; however, at the genus level, patients with chronic insomnia exhibited a significantly higher relative abundance of *Eggerthella*. The reason for this can be attributed to the fact that both groups of patients had a similar range of diseases, except for the chronic insomnia symptoms.

PICRUSt algorithm was employed to predict the functional profiles of gut microbiota based on the predicted metagenome. Subsequently, Kyoto Encyclopedia of Genes and Genomes (KEGG) pathway functions were categorized using PICRUSt. The findings from the PICRUSt analysis revealed significant reductions in styrene degradation and aminobenzoate degradation pathways among patients with chronic insomnia. The metabolic pathways, including riboflavin metabolism, cofactors and vitamins metabolism, nitrotoluene degradation, ethylbenzene degradation, chlorocyclohexane and chlorobenzene degradation, exhibit diversity. Although no statistically significant difference was observed (*p* value ranging from 0.0516 to 0.0616), this could be attributed to the limited number of patients enrolled in this study.

### The regulatory effects of FMT on gut microbiota in patients with chronic insomnia

**Figure 6:**
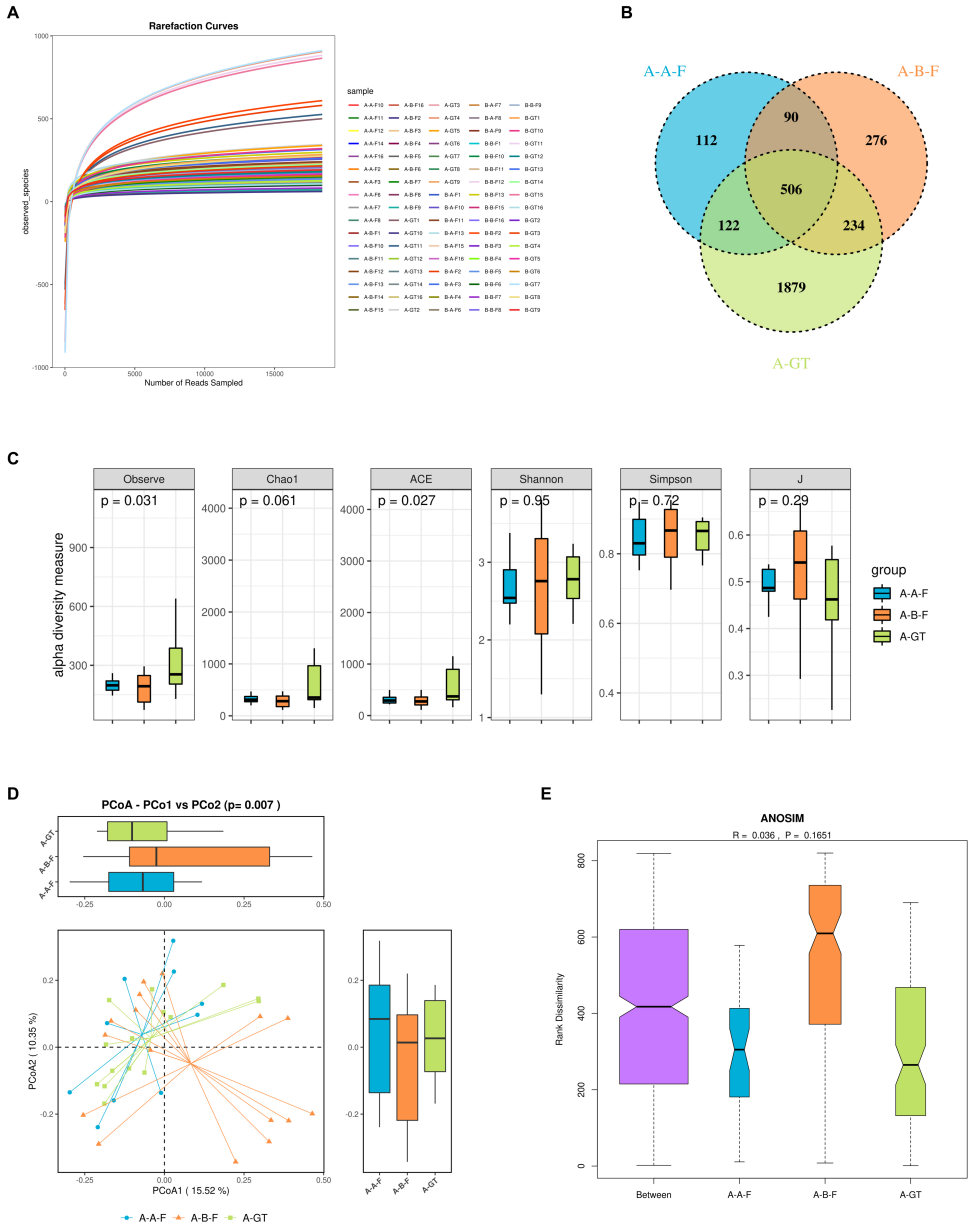
Stool microbiota composition analysis the healthy donors (marked as A-GT), chronic insomnia patients (marked as A-B-F) and patients post FMT treatment (marked as A-A-F). **(A)** The rarefaction curve of the current sequencing data. **(B)** Venn diagram indicating the number of differential OTUs in each group. **(C)** Alpha diversity index box chart, the abscissa represents sample grouping, and the ordinate is the alpha index. **(D)** Principal coordinate analysis (PCoA) of the gut microbiota among the healthy donors, chronic insomnia patients and patients post FMT treatment. The distance between the samples represents the similarity of the gut microbiota composition, and a closer distance indicates higher similarity. **(E)** Analysis of similarities (ANOSIM) among the healthy donors, chronic insomnia patients and patients post FMT treatment. A-GT: healthy donors, A-B-F: Patients with chronic insomnia before FMT intervention, A-A-F: Patients with chronic insomnia after FMT intervention.

Based on the observed plateau in the rarefaction curve of the current sequencing data suggested that sequencing depth was enough to capture all bacterial species across all samples and sufficient for downstream analysis (Figure 6A). Venn diagram of gut microbial species among the groups of pre- and post-FMT therapy, as well as healthy donors was shown in Figure 6B. The alpha diversity indices such as observe and ACE showed significant difference between the healthy donors and the insomnia groups, while no notable disparity was observed in the diversity indexes (Shannon and Simpson, Figure 6C). This suggested that insomnia disorder may result in alteration of gut microbiota diversity. The β-diversity revealed substantial structural disparities between insomnia patients pre- and post-FMT therapy, as well as healthy donors. PCoA analysis unveiled a substantial divergence in the composition of gut microbiota between individuals with insomnia and healthy donors. Following FMT treatment, the dysbiosis of the gut microbiota was significantly ameliorated with no apparent separation from that observed among healthy donors (Figure 6D). Compared to healthy donors, patients with insomnia exhibited dysbiosis of gut microbiota, characterized by a significant increase in the relative abundance of *Howardella* at the general level. The ANOSIM analysis based on the Bray Curtis distance demonstrated a statistically significant disparity in gut microbiota composition between the baseline and post-FMT treatment groups (R > 0), although no significant differences were observed in terms of group contributions (*p* = 0.1651). The findings suggest that FMT effectively ameliorates dysbiosis in the gut microbiota of insomnia patients, resulting in a microbial community resembling that of healthy donors (Figure 6E).

**Figure 7:**
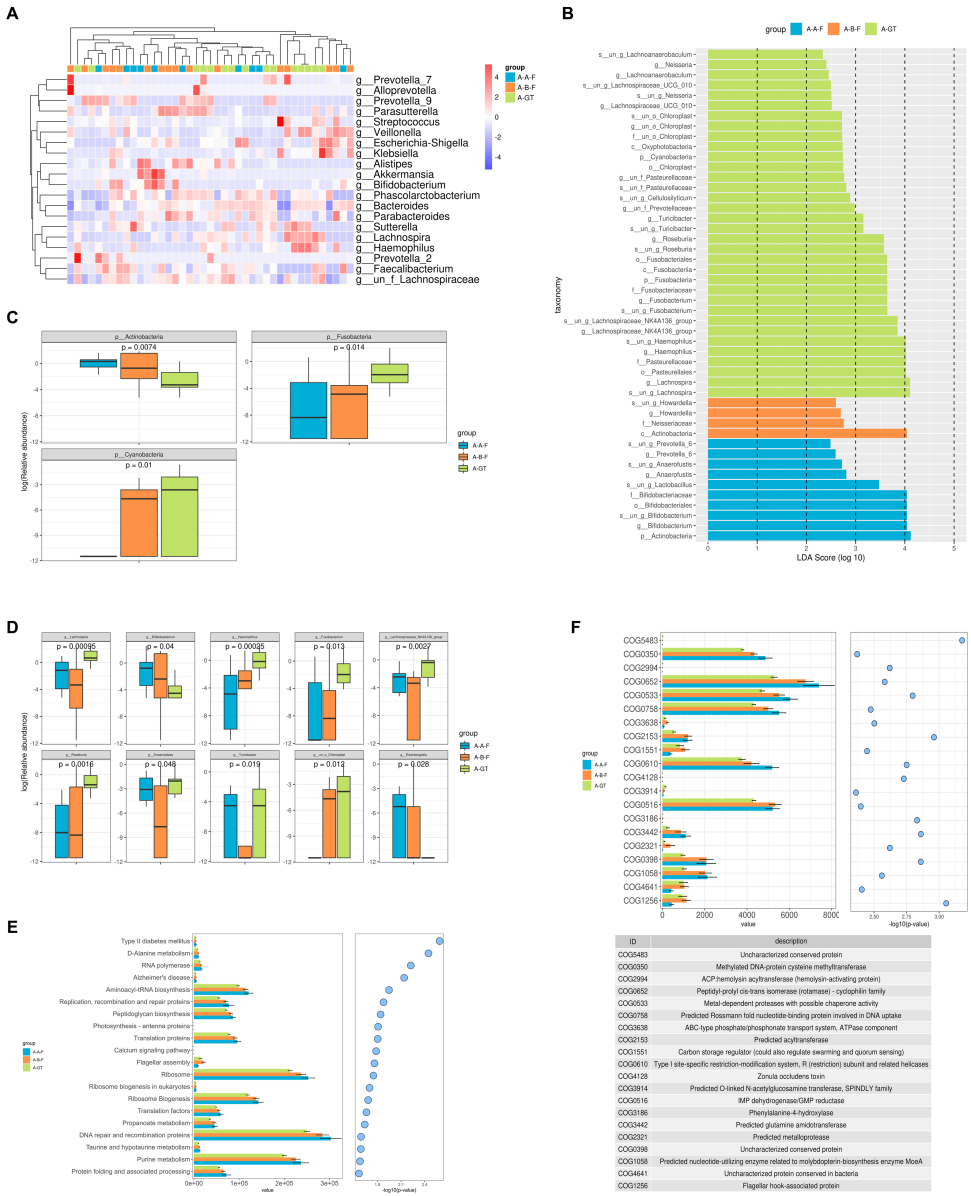
Histogram of taxonomic profiles of the gut microbiota among the healthy donors (marked as A-GT), chronic insomnia patients (marked as A-B-F) and patients post FMT treatment (marked as A-A-F). **(A)** Heat maps of different bacteria at genus level in the healthy donor, chronic insomnia patients before and after FMT treatment. **(B)** LDA scores of different species of gut microbiota in the healthy donor, chronic insomnia patients before and after FMT treatment. **(C)** Analysis on the difference of the gut microbiota at the phylum level. **(D)** Analysis on the difference of the gut microbiota at the genus level. **(E)** Kyoto Encyclopedia of Genes and Genomes (KEGG) pathway functions analyzes. **(F)** COG analyzes.

The LEfSe analysis was employed to ascertain the dominant flora, with an LDA value >2 indicating a statistically significant difference in the healthy donor, chronic insomnia patients before and after FMT treatment. The results revealed a significant increase in the relative abundance of *Lactobacillus*, *Bifidobacterium, Prevotella_6*, and *Anaerofustis* at the genus level within the intestinal tracts of insomnia patients after FMT treatment (Figure 7B). Analysis on the difference of the gut microbiota at the phylum level among the healthy donors, pre and post FMT treatment group showed that *Actinobacteria, Fusobacteria* and *Cyanobacteria* displayed marked differences. The relative abundance of *Actinobacteria* in the gut was significantly increased, while *Cyanobacteria* showed a significant decrease in chronic insomnia patients who underwent FMT treatment (Figure 7C). The top 20 genera with significant differences in the intestinal flora at the genus level in chronic insomnia patients before and after FMT treatment was illustrated in Figure 7A. Additionally, Figure 7D presents a box diagram showcasing the top 10 genera with notable variations, we found that FMT treatment can significantly enhance the relative abundance of *Lactobacillus, Bifidobacterium, Turicibacter, Anaerostipes*, and *Eisenbergiella* in the gastrointestinal tract of patients with chronic insomnia.

To further elucidate the underlying mechanism of gut microbiota and metabolites in the treatment of insomnia, we conducted a comprehensive KEGG analysis. The PICRUSt analysis results revealed significant alterations in 40 KEGG pathways among insomnia patients before and after FMT treatment, as well as healthy donors, especially the D-Alanine metabolism, purine metabolism, purine metabolism, taurine and hypotaurine metabolism, DNA repair and recombination proteins, protein folding and associated processing, replication, recombination and repair proteinsribosome biogenesis, peptidoglycan biosynthesis aminoacyl-tRNA biosynthesis, translation proteins were marked different among the groups (Figure 7E).

### Correlation analysis of different flora with sleep quality, mood and quality of life scores

**Figure 8:**
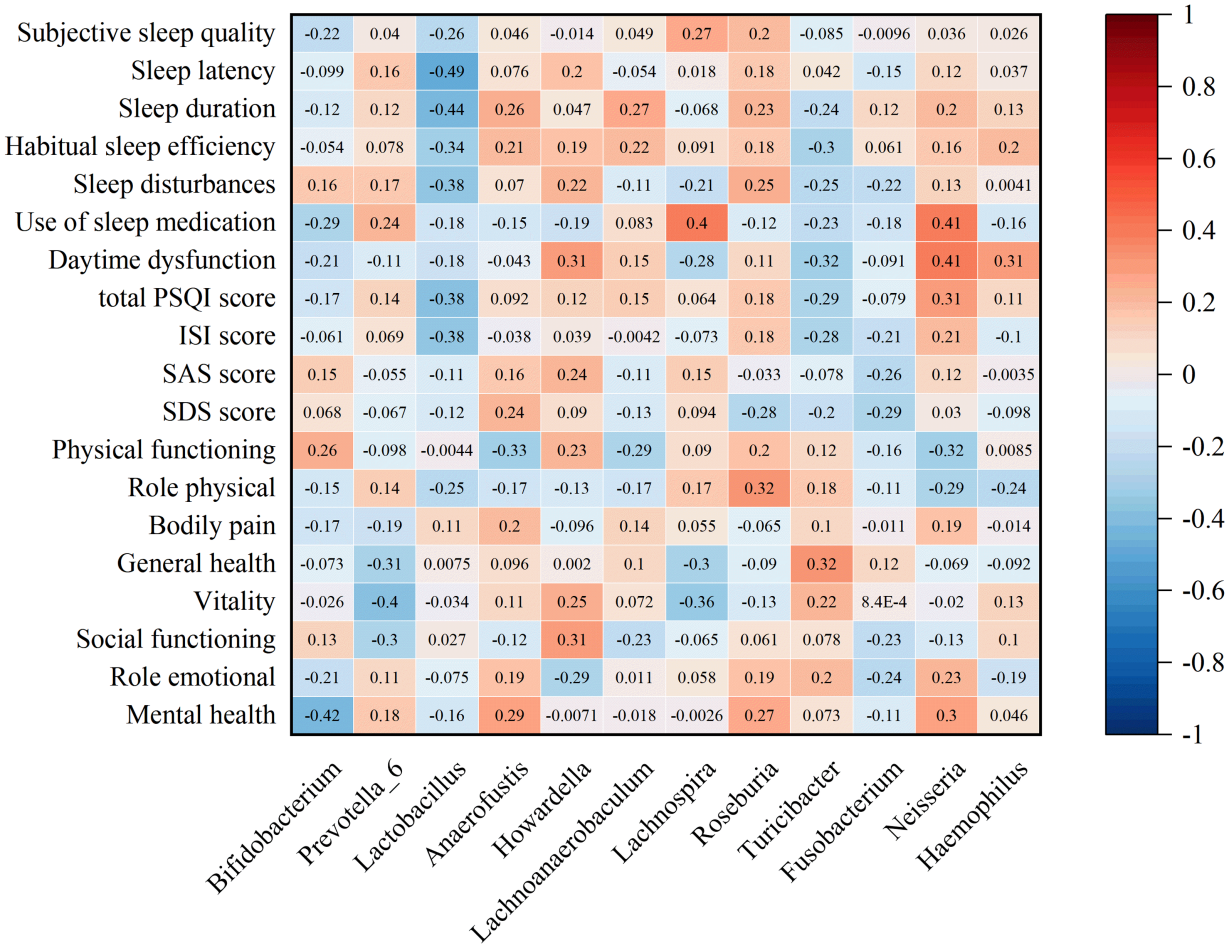
Correlation analysis of different species of gut microbiota in insomnia patients before and after FMT treatment.

The correlation analysis of the top 20 bacterial types with significant differences at the genus level and scores of sleep quality, mood, and quality of life in patients with chronic insomnia before and after FMT treatment revealed a negative correlation between *Bifidobacterium*, *Lactobacillus*, *Turicibacter*, and *Fusobacterium*. Conversely, a positive correlation was observed with *Prevotella_6*, *Anaerofustis*, *Howardella*, *Lachnoanaerobaculum*, *Lachnospira*, *Roseburia*, *Neisseria,* and *Haemophilus*. Furthermore, *Bifidobacterium*, *Lactobacillus* and *Fusobacterium* exhibited negative correlations with ISI scores, PQSI total scores, PQSI subitem scores and SF-36 subitem scores in patients suffering from chronic insomnia (Figure 8).

## Discussion

Sleep disorders have become an epidemic, whereas chronic sleep disturbances cause long-lasting gastrointestinal, psychiatric, and neurological problems. A total of 33 subjects diagnosed with chronic diseases were enrolled in this study, 51.5% (17/33) of them had comorbid chronic insomnia, and the prevalence rate of insomnia was found to be significantly higher among women compared to men. The high prevalence of chronic insomnia in this study is considered to be associated with the inclusion of patients suffering from chronic diseases (including rCDI, UC, IBS, chronic consitpaiton and Parkinson disease). Insomnia is comorbid with a range of psychiatric and inflammatory disorders as well as metabolic syndromes. Individuals with IBS have a twofold higher risk of comorbid depression, anxiety, and sleep disorders, conversely, patients presenting with depression and anxiety are reported to have a heightened susceptibility to developing IBS (Liu et al., 2023). Idiopathic rapid eye movement sleep behavior disorder is a powerful early sign of Parkinson’s disease (Postuma et al., 2019). The available evidences suggest that individuals with chronic lifelong diseases are at a heightened vulnerability to sleep disorders. The co-existence of chronic disease with psychiatric disorders can be a big burden, and the presence of comorbid depression exerts a more negative effect on health than depression or anxiety does alone (Moussavi et al., 2007). The growing prevalence of sleep disorders has raised the question of what governs sleep quality and how these factors can be manipulated to improve sleep (Sen et al., 2021).

Accumulated evidences suggest that the gut microbiota plays a crucial role in regulating human health, particularly metabolic, immunological, and neurobehavioral functions. The strong correlation between psychiatric disorders, including sleep, and dysbiosis of gut microbiota or alterations in intestinal metabolism has been extensively discussed, indicating an inherent cross-link between the gut microbiota and brain regions that has been referred to as the microbiota-gut-brain axis. In 1983, Rechtschaffen and colleagues conducted groundbreaking research in the fields of sleep, gut microbiota, and health. Their findings revealed a systemic deterioration in rats exposed to prolonged sleep deprivation (Rechtschaffen et al., 1983). Benedict and colleagues provide the inaugural published insights into the intricate interplay between sleep and gut microbiota in human subjects, thereby introducing a pioneering exploration of acute sleep restriction’s impact on gut microbiota. This initiates a crucial discourse on future investigations of the gut microbiota in human sleep research (Benedict et al., 2016). A recent murine study revealed that mice with depleted gut bacteria using antibiotics exhibited significant sleep disturbances, characterized by spending less time in non-REM sleep during the light phase and spending more time in non-REM and REM sleep during the dark phase, leading to poorer quality of sleep (Ogawa et al., 2020). Disrupted sleep can also induce alterations in the composition of the gut microbiome. In human male adults, the sleep efficiency and total sleep time were positively correlated with the diversity of the gut microbiota (Smith et al., 2019). Compared to healthy controls, insomnia patients exhibit reduced microbial richness and diversity in the gut microbiota, as well as a depletion in anaerobic bacteria and short-chain fatty acid (SCFA)-producing bacteria. Moreover, there is an expansion of potential pathobionts present (Li et al., 2020). Despite the ongoing lack of understanding regarding the precise pathological mechanisms underlying insomnia, there is a widespread recognition that maintaining gut microbiota homeostasis plays a pivotal role in determining both the onset and severity of this disorder. Moreover, insomnia has been shown to exert profound structural and functional effects on the gut microbiota.

Here we investigate characteristics of fecal microbiome diversity and composition in patients with chronic bowel disease comorbid chronic insomnia or not. The analysis of gut microbiota characteristics in the two groups at baseline (before FMT treatment) revealed no significant differences at the phylum level. However, a statistically significant increase in the relative abundance of *Eggerthella* genus was observed among patients with chronic insomnia compared to individuals without insomnia. The observed dissimilarities at the phylum and genus levels between the two groups are hypothesized to be associated with their similar disease spectrum. The bacterium *Eggerthella* is a gram-positive, anaerobic, non-sporulating microorganism that typically resides in the human microbiota. However, emerging evidence suggests its potential as a significant human pathogen capable of causing life-threatening infections under specific circumstances. Additionally, *Eggerthella* is significantly enriched in patients with various autoimmune diseases and exhibits an increased presence in individuals with inflammatory bowel disease (IBD) and exacerbates colitis models (Alexander et al., 2022). Interestingly, a recent meta-analysis of psychiatric disorders and gut microbiota showed that enriched levels of *Eggerthella* were consistently shared between major depressive disorder, anxiety, bipolar disorder, psychosis and schizophrenia, suggesting these disorders are characterized by an increase of pro-inflammatory genera (Nikolova et al., 2021). The bulk of earlier research into insomnia on gut microbiota has focused on animal models of sleep deprivation and intervention studies involving probiotics. To the best of our knowledge, our results represent the first evidence linking *Eggerthella* to chronic insomnia in humans, as revealed by a comprehensive real-world case analysis. However, sleep is a complex physiological process of the body, the initiation and maintenance of the sleep–wake cycle are regulated by both the central nervous system and peripheral tissues. The understanding of *Eggerthella’s* pathogenic mechanism and treatment options still requires further enhancement. The acquisition of a substantial amount of clinical data is essential for a comprehensive exploration in this field.

The findings from the PICRUSt analysis revealed significant reductions in styrene degradation and aminobenzoate degradation pathways among patients with with refractory bowel disease comorbid chronic insomnia. Although no statistically significant differences were observed among the metabolic pathways, including riboflavin metabolism, cofactors and vitamins metabolism, nitrotoluene degradation, ethylbenzene degradation, chlorocyclohexane and chlorobenzene degradation; however, they did exhibit variations, which maybe due to the limited sample size. These findings may have implications for future investigations into the underlying mechanism of chronic insomnia, as the study included various disease types but shared a common comorbidity: chronic insomnia. For example, there were significant relationships between moderate/severe insomnia and the presence of malnutrition and risk of malnutrition (Soysal et al., 2019). The regression models showed that patients with generalized anxiety disorder displaying low levels of vitamin D demonstrate a poor subjective quality of sleep and heightened anxiety. A strong relationship between peripheral biomarkers of calcium homeostasis imbalance, insomnia, poor sleep quality, and anxiety symptomatology was underlined (Carbone et al., 2023).

Gaining insights into the correlation between sleep and gut microbiota may facilitate the development of targeted interventions aimed at enhancing sleep health by modulating the composition of gut microbiota. Gut microbiota-targeted interventions have been shown to exert a therapeutic effect on sleep disturbances. In 2013, Dinan and colleagues defined the term “psychobiotics” as a novel class of probiotics that suggest potential applications in treating psychiatric diseases (Dinan et al., 2013). Psychobiotics are efficacious in ameliorating neurodegenerative and neurodevelopmental disorders, including autism spectrum disorder (ASD), Parkinson’s disease (PD) and Alzheimer’s disease (AD), which led researchers to focus on a new area in neuroscience (Cheng et al., 2019). Evidence in preschool-aged children indicates a correlation between sleep duration and the diversity of gut microbiota, with children who have longer total nighttime sleep showing a higher relative abundance of *Bifidobacterium* (Wang et al., 2022). Importantly, studies conducted in adult humans have also demonstrated the efficacy of *Bifidobacterium*, whether administered as a standalone probiotic or in combination with other probiotics, in modulating mood (Pinto-Sanchez et al., 2017). Daily administration of *Lactobacillus plantarum* may lead to a decrease in depressive symptoms, fatigue level, cortical excitation, and an improvement in sleep quality during the deep sleep stage (Ho et al., 2021). In addition, lower *Bifidobacterium* and/or *Lactobacillus* counts were observed in subjects with major depressive disorder, probiotics composed of specific strains of genera *Lactobacillus* and *Bifidobacterium* have the potential to prevent and treat various psychiatric conditions such as depression and anxiety (Cheng et al., 2019).

FMT is a robust method to rebalance the gut microbiota ecosystem of recipient through the transfer of healthy donor-derived gut microbiota to patients. Engraftment of donor microbiota resulted in a long-lasting response in patients with recurrent *Clostridium difficile* infection and active ulcerative colitis (Weingarden et al., 2015; Fang et al., 2021). Furthermore, FMT is a well-tolerated, effective, and safe method as an add-on therapy in patients with mild-to-moderate ulcerative colitis (Tkach et al., 2023). In the present study, we demonstrated that FMT significantly enhanced sleep quality, alleviated anxiety and depression symptoms, and improved the quality of life in individuals with chronic insomnia as assessed through self-reported questionnaires. The ISI and PSQI score sub-item indicates that FMT significantly improves insomnia by reducing sleep onset latency, enhancing sleep efficiency, and subsequently alleviating symptoms of daytime fatigue. It is encouraging to note that patients without insomnia who underwent FMT also reported an improvement in their sleep quality. The application of FMT can also enhance the psychological well-being of patients with chronic insomnia, alleviating symptoms of anxiety and depression, while concurrently enhancing their quality of life. The analysis of gut microbiota before and after FMT treatment revealed a significant improvement in dysbiotic gut microbiota among patients with chronic insomnia who underwent FMT, leading to a composition resembling that of healthy donors, indicating a clear restoration in the dysbiosis of gut microbiota. The LEfSe analysis found that there was a significant increase in the relative abundance of *Lactobacillus*, *Bifidobacterium, Prevotella_6*, and *Anaerofustis* at the genus level within their gut microbiota. The correlation analysis revealed a significant positive correlation between the efficacy of FMT in treating insomnia and the presence of *Lactobacillus* and *Bifidobacterium*. The findings indicated that FMT exhibited the potential to enhance the abundance of probiotics in the gut of individuals suffering from chronic insomnia. The majority of these taxa have been linked with sleep-related neurochemicals in previous studies, such as serotonin, tryptophan and gamma-aminobutyric acid. For example, *Lactobacillus* and *Bifidobacterium* produce gamma-aminobutyric acid (GABA) and serotonin. In addition, strains of *Lactobacillus* produce acetylcholine (Cheng et al., 2019). Additionally, *Bacteroides* is a crucial taxon involved in the production of the SCFA propionate through the succinate pathway (Louis and Flint, 2017).

However, unlike probiotics, which only contain a limited number of bacterial species, FMT contains thousands of species native to the human gut. On the other hand, apart from alterations in the composition of the gut microbiota, disruptions were observed in both signaling pathways and metabolic functions among individuals with insomnia disorder. Mice with depleted gut bacteria displayed impaired sleep quality and decreased concentrations of serotonin in their gut (Ogawa et al., 2020). Following FMT from sleep-fragmented mice, the recipients mimicked the donor’s characteristics, including increased systemic white adipose tissue inflammation and altered insulin sensitivity (Poroyko et al., 2016). These suggest that FMT is likely to exert its therapeutic effects through multiple mechanisms. In the present study, PICRUSt supported the gut taxa composition, signaling pathways, and metabolic functions perturbed by insomnia disorder. There were significant alterations observed in 40 KEGG pathways among insomnia patients before and after FMT treatment, as well as healthy donors. Specifically, the D-alanine metabolism, purine metabolism, taurine and hypotaurine metabolism, DNA repair and recombination proteins, protein folding and associated processing, replication, recombination and repair proteins ribosome biogenesis, peptidoglycan biosynthesis aminoacyl-tRNA biosynthesis, translation proteins exhibited marked differences among the groups. This implies the potential mediating roles of neurotransmitters in connecting gut microbiota and sleep (Fung et al., 2019).

Our study demonstrates the following characteristics: firstly, it was conducted in a real-world setting and encompassed a diverse range of medical conditions, including rCDI, IBS, chronic constipation, UC, and Parkinson’s disease with chronic constipation and all patients received a donor-recipient matching FMT tailored to these diseases. Additionally, a comparison was conducted between the gut microbiota characteristics of chronic disease patients with and without comorbid chronic insomnia at baseline. This makes it more applicable in clinical settings regarding FMT for the treatment of chronic insomnia. Secondly, there is a distinction in the definition of insomnia, using the ISI and PSQI for screening, grouping, and efficacy assessment. We define chronic insomnia as having a PSQI score equal to or exceeding 11, which provided evidence supporting the therapeutic potential of FMT in the treatment of chronic insomnia. Thirdly, we conducted an analysis on the characteristics of gut microbiota of chronic insomnia patients both before and after FMT treatment, as well as its correlation with treatment efficacy.

Of course, the current study is merely a limited sample size observational study. In addition, this study was primarily relied on self-reported questionnaires and lacked actigraphy sleep measures included total nighttime sleep (TST), sleep efficiency (SE), and wake-time after sleep onset (WASO). Therefore, it is imperative to conduct large-scale randomized controlled trials in conjunction with metagenomic and metabolomic to identify the effects of FMT on chronic insomnia. Although the potential benefits of FMT in various diseases, there exists a variation and complexity in procedural agreement among research groups for performing FMT, and the optimal approach to achieve engraftment after FMT remains unclear (Bokoliya et al., 2021). The adoption of the use of antibiotics prior to FMT and data remain controversial (Cammarota et al., 2017; Ng et al., 2020). As known, the host-microbiota encompasses a variety of microbial communications, including the oral, nasal, lung, gut, skin, bladder, and vaginal microbiota. Microbiota in one organ have the potential to influence those in another, and they can interact with the gut microbiota via inflammatory and immune system pathways (Hashimoto, 2023). The oral cavity serves as the primary entry point to the human body, and the oral microbiota represents the second-largest microbial community in humans, which may potentially contribute to the development and progression of psychiatric and neurodegenerative disorders, as well as autoimmune diseases. Patients with chronic insomnia harbored a significantly higher diversity of oral bacteria when compared to healthy controls. More importantly, the results revealed that the diversity and relative abundance of the bacterial community was significantly altered among different tongue coatings in patients but not in healthy individuals (Liu et al., 2020). Compared to the control group, insomniac patients exhibited a significantly higher abnormal low-frequency/high-frequency (LF/HF) ratio. Furthermore, linear discriminant analysis of oral microbial population revealed that the relative abundances of *Clostridia, Veillonella, Bacillus* and *Lachnospiraceae* were significantly higher in the insomniac patients than the control group (Park et al., 2022). The impact of various lifestyle and environmental factors on sleep is well-documented. Increasingly, evidence suggests that gut microbiota plays a significant role in regulating brain function and behavior (Sen et al., 2021). The present study included individuals with underlying chronic conditions who mainly reported no history of smoking or alcohol consumption, and their lifestyle and dietary practices aligned with widely promoted eating patterns prevalent in Anhui Province, China. Considering the exploratory nature of this study and the preliminary positive findings of FMT in managing adults chronic insomnia, it is imperative to incorporate the potential influence and significance of lifestyle factors and antibiotic pretreatment prior to FMT in subsequent randomized controlled trials (RCTs).

## Conclusion

The present study represents the first real-world case analysis demonstrating that *Eggerthella* could potentially serve as a distinctive genus associated with chronic insomnia. FMT therapy not only improves sleep quality, but also enhances antidepressant and anxiolytic effects, as well as overall quality of life in patients with chronic insomnia and the effects was positive associated with the augmentation of probiotic microorganisms, such as *Bifidobacterium*, *Lactobacillus*, *Turicibacter*, and *Fusobacterium*. Our preliminary empirical work supported the acceptability and feasibility of using FMT to be a novel treatment option for adults with chronic insomnia and provide an alternative to traditional treatments for insomnia., the mechanism may be correlated with reshaping the dysbiosis of gut microbiota and increasing the abundance of beneficial bacteria such as *Bifidobacterium* and *Lactobacillus*.

## Data availability statement

The datasets presented in this study can be found in online repositories. The names of the repository/repositories and accession number(s) can be found at: https://www.ncbi.nlm.nih.gov/, PRJNA1020211.

## Ethics statement

The studies involving humans were approved by the Ethics Committee of the Second Affiliated Hospital of Anhui Medical University. The studies were conducted in accordance with the local legislation and institutional requirements. The participants provided their written informed consent to participate in this study.

## Author contributions

HF: Conceptualization, Data curation, Formal analysis, Funding acquisition, Investigation, Methodology, Project administration, Resources, Software, Supervision, Validation, Visualization, Writing – original draft, Writing – review & editing. TY: Data curation, Formal analysis, Funding acquisition, Investigation, Methodology, Resources, Software, Validation, Visualization, Writing – review & editing. WL: Data curation, Formal analysis, Methodology, Resources, Validation, Visualization, Writing – review & editing. NP: Data curation, Formal analysis, Investigation, Methodology, Validation, Visualization, Writing – review & editing. HX: Data curation, Formal analysis, Methodology, Writing – review & editing. QZ: Data curation, Formal analysis, Investigation, Methodology, Visualization, Writing – review & editing. YS: Data curation, Formal analysis, Investigation, Methodology, Validation, Visualization, Writing – review & editing. KX: Data curation, Formal analysis, Investigation, Methodology, Visualization, Writing – review & editing. JW: Conceptualization, Data curation, Formal analysis, Funding acquisition, Investigation, Methodology, Project administration, Resources, Supervision, Validation, Visualization, Writing – review & editing.

## References

[ref1] Abenavoli L. Maurizi V. Rinninella E. Tack J. di Berardino A. Santori P.. (2022). Fecal microbiota transplantation in NAFLD treatment. Medicina (Kaunas) 58:1559. doi: 10.3390/medicina58111559, PMID: 36363516 PMC9695159

[ref2] Abenavoli L. Scarlata G. G. M. Paravati M. R. Boccuto L. Luzza F. Scarpellini E. (2023). Gut microbiota and liver transplantation: immune mechanisms behind the rejection. Biomedicine 11:1792. doi: 10.3390/biomedicines11071792, PMID: 37509432 PMC10376769

[ref3] Alexander M. Ang Q. Y. Nayak R. R. Bustion A. E. Sandy M. Zhang B.. (2022). Human gut bacterial metabolism drives Th17 activation and colitis. Cell Host Microbe 30, 17–30.e9. doi: 10.1016/j.chom.2021.11.001, PMID: 34822777 PMC8785648

[ref4] Benedict C. Vogel H. Jonas W. Woting A. Blaut M. Schürmann A.. (2016). Gut microbiota and glucometabolic alterations in response to recurrent partial sleep deprivation in normal-weight young individuals. Mol Metab 5, 1175–1186. doi: 10.1016/j.molmet.2016.10.003, PMID: 27900260 PMC5123208

[ref5] Besedovsky L. Lange T. Haack M. (2019). The sleep-immune crosstalk in health and disease. Physiol. Rev. 99, 1325–1380. doi: 10.1152/physrev.00010.2018, PMID: 30920354 PMC6689741

[ref6] Bokoliya S. C. Dorsett Y. Panier H. Zhou Y. (2021). Procedures for Fecal microbiota transplantation in murine microbiome studies. Front. Cell. Infect. Microbiol. 11:711055. doi: 10.3389/fcimb.2021.711055 34621688 PMC8490673

[ref7] Bongiorno D. Bivona D. A. Cicino C. Trecarichi E. M. Russo A. Marascio N.. (2023). Omic insights into various ceftazidime-avibactam-resistant *Klebsiella pneumoniae* isolates from two southern Italian regions. Front. Cell. Infect. Microbiol. 12:1010979. doi: 10.3389/fcimb.2022.1010979, PMID: 36683697 PMC9851273

[ref8] Cammarota G. Ianiro G. Tilg H. Rajilić-Stojanović M. Kump P. Satokari R.. (2017). European consensus conference on faecal microbiota transplantation in clinical practice. Gut 66, 569–580. doi: 10.1136/gutjnl-2016-313017, PMID: 28087657 PMC5529972

[ref9] Carbone E. A. Menculini G. de Filippis R. D’Angelo M. de Fazio P. Tortorella A.. (2023). Sleep disturbances in generalized anxiety disorder: the role of calcium homeostasis imbalance. Int. J. Environ. Res. Public Health 20:4431. doi: 10.3390/ijerph20054431, PMID: 36901441 PMC10002427

[ref10] Cheng L. H. Liu Y. W. Wu C. C. Wang S. Tsai Y. C. (2019). Psychobiotics in mental health, neurodegenerative and neurodevelopmental disorders. J. Food Drug Anal. 27, 632–648. doi: 10.1016/j.jfda.2019.01.002, PMID: 31324280 PMC9307042

[ref11] Cox L. M. Schafer M. J. Sohn J. Vincentini J. Weiner H. L. Ginsberg S. D.. (2019). Calorie restriction slows age-related microbiota changes in an Alzheimer's disease model in female mice. Sci. Rep. 9:17904. doi: 10.1038/s41598-019-54187-x, PMID: 31784610 PMC6884494

[ref12] Cryan J. F. O'Riordan K. J. Sandhu K. Peterson V. Dinan T. G. (2020). The gut microbiome in neurological disorders. Lancet Neurol. 19, 179–194. doi: 10.1016/S1474-4422(19)30356-4 31753762

[ref13] Dinan T. G. Stanton C. Cryan J. F. (2013). Psychobiotics: a novel class of psychotropic. Biol. Psychiatry 74, 720–726. doi: 10.1016/j.biopsych.2013.05.001, PMID: 23759244

[ref14] Dos Reis L. L. Terra Loyola V. Leopoldino de Bortolli C. Levy Andersen M. Tufik S. Hachul H. (2021). Effects of supplementation with lactobacillus probiotics on insomnia treatment. Altern. Ther. Health Med. 27, 178–184. 33609341

[ref15] Fang H. Fu L. Li X. Lu C. Su Y. Xiong K.. (2021). Long-term efficacy and safety of monotherapy with a single fresh fecal microbiota transplant for recurrent active ulcerative colitis: a prospective randomized pilot study. Microb. Cell Factories 20:18. doi: 10.1186/s12934-021-01513-6, PMID: 33468164 PMC7816432

[ref16] Fang H. Fu L. Wang J. (2018). Protocol for Fecal microbiota transplantation in inflammatory bowel disease: a systematic review and meta-analysis. Biomed. Res. Int. 2018, 8941340–8941311. doi: 10.1155/2018/8941340, PMID: 30302341 PMC6158944

[ref17] Fung T. C. Vuong H. E. Luna C. D. G. Pronovost G. N. Aleksandrova A. A. Riley N. G.. (2019). Intestinal serotonin and fluoxetine exposure modulate bacterial colonization in the gut. Nat. Microbiol. 4, 2064–2073. doi: 10.1038/s41564-019-0540-4 31477894 PMC6879823

[ref18] Han M. Yuan S. Zhang J. (2022). The interplay between sleep and gut microbiota. Brain Res. Bull. 180, 131–146. doi: 10.1016/j.brainresbull.2021.12.016, PMID: 35032622

[ref19] Hashimoto K. (2023). Emerging role of the host microbiome in neuropsychiatric disorders: overview and future directions. Mol. Psychiatry. doi: 10.1038/s41380-023-02287-6, PMID: 37845499 PMC10730413

[ref20] Ho Y. T. Tsai Y. C. Kuo T. B. J. Yang C. C. H. (2021). Effects of *Lactobacillus plantarum* PS128 on depressive symptoms and sleep quality in self-reported insomniacs: a randomized, double-blind, Placebo-Controlled Pilot Trial. Nutrients 13:2820. doi: 10.3390/nu13082820, PMID: 34444980 PMC8402034

[ref21] Irwin C. McCartney D. Desbrow B. Khalesi S. (2020). Effects of probiotics and paraprobiotics on subjective and objective sleep metrics: a systematic review and meta-analysis. Eur. J. Clin. Nutr. 74, 1536–1549. doi: 10.1038/s41430-020-0656-x, PMID: 32433598

[ref22] Koopman A. D. M. Beulens J. W. Dijkstra T. Pouwer F. Bremmer M. A. van Straten A.. (2020). Prevalence of insomnia (symptoms) in T2D and association with metabolic parameters and Glycemic control: meta-analysis. J. Clin. Endocrinol. Metab. 105, 614–643. doi: 10.1210/clinem/dgz065, PMID: 31603475 PMC7110921

[ref23] Kurokawa S. Kishimoto T. Mizuno S. Masaoka T. Naganuma M. Liang K. C.. (2018). The effect of fecal microbiota transplantation on psychiatric symptoms among patients with irritable bowel syndrome, functional diarrhea and functional constipation: an open-label observational study. J. Affect. Disord. 235, 506–512. doi: 10.1016/j.jad.2018.04.038, PMID: 29684865

[ref24] Li Y. Zhang B. Zhou Y. Wang D. Liu X. Li L.. (2020). Gut microbiota changes and their relationship with inflammation in patients with acute and chronic insomnia. Nat. Sci. Sleep 12, 895–905. doi: 10.2147/NSS.S271927, PMID: 33177907 PMC7652227

[ref25] Liu B. Lin W. Chen S. Xiang T. Yang Y. Yin Y.. (2019). Gut microbiota as an objective measurement for auxiliary diagnosis of insomnia disorder. Front. Microbiol. 10:1770. doi: 10.3389/fmicb.2019.01770, PMID: 31456757 PMC6701205

[ref26] Liu M. Wang X. Wu F. Dai N. Chen M. Yu J.. (2020). Variations of Oral microbiome in chronic insomnia patients with different tongue features. Am. J. Chin. Med. 48, 923–944. doi: 10.1142/S0192415X20500445, PMID: 32436424

[ref27] Liu M. Ye Z. Wu Q. Yang S. Zhang Y. Zhou C.. (2023). Healthy sleep, mental health, genetic susceptibility, and risk of irritable bowel syndrome. J. Affect. Disord. 331, 25–32. doi: 10.1016/j.jad.2023.03.033, PMID: 36934852

[ref28] Louis P. Flint H. J. (2017). Formation of propionate and butyrate by the human colonic microbiota. Environ. Microbiol. 19, 29–41. doi: 10.1111/1462-2920.13589 27928878

[ref29] Moussavi S. Chatterji S. Verdes E. Tandon A. Patel V. Ustun B. (2007). Depression, chronic diseases, and decrements in health: results from the world health surveys. Lancet 370, 851–858. doi: 10.1016/S0140-6736(07)61415-9, PMID: 17826170

[ref30] Ng S. C. Kamm M. A. Yeoh Y. K. Chan P. K. S. Zuo T. Tang W.. (2020). Scientific frontiers in faecal microbiota transplantation: joint document of Asia-Pacific Association of Gastroenterology (APAGE) and Asia-Pacific Society for Digestive Endoscopy (APSDE). Gut 69, 83–91. doi: 10.1136/gutjnl-2019-319407, PMID: 31611298 PMC6943253

[ref31] Nikolova V. L. Smith M. R. B. Hall L. J. Cleare A. J. Stone J. M. Young A. H. (2021). Perturbations in gut microbiota composition in psychiatric disorders: a review and meta-analysis. JAMA Psychiatry 78, 1343–1354. doi: 10.1001/jamapsychiatry.2021.2573, PMID: 34524405 PMC8444066

[ref32] Ogawa Y. Miyoshi C. Obana N. Yajima K. Hotta-Hirashima N. Ikkyu A.. (2020). Gut microbiota depletion by chronic antibiotic treatment alters the sleep/wake architecture and sleep EEG power spectra in mice. Sci. Rep. 10:19554. doi: 10.1038/s41598-020-76562-9, PMID: 33177599 PMC7659342

[ref33] Park S. H. Shin N. R. Yang M. Bose S. Kwon O. Nam D. H.. (2022). A clinical study on the relationship among insomnia, tongue diagnosis, and Oral microbiome. Am. J. Chin. Med. 50, 773–797. doi: 10.1142/S0192415X2250032X, PMID: 35380093

[ref34] Pinto-Sanchez M. I. Hall G. B. Ghajar K. Nardelli A. Bolino C. Lau J. T.. (2017). Probiotic *Bifidobacterium longum* NCC3001 reduces depression scores and alters brain activity: a pilot study in patients with irritable bowel syndrome. Gastroenterology 153, 448–459.e8. doi: 10.1053/j.gastro.2017.05.003, PMID: 28483500

[ref35] Poroyko V. A. Carreras A. Khalyfa A. Khalyfa A. A. Leone V. Peris E.. (2016). Chronic sleep disruption alters gut microbiota, induces systemic and adipose tissue inflammation and insulin resistance in mice. Sci. Rep. 6:35405. doi: 10.1038/srep35405, PMID: 27739530 PMC5064361

[ref36] Postuma R. B. Iranzo A. Hu M. Högl B. Boeve B. F. Manni R.. (2019). Risk and predictors of dementia and parkinsonism in idiopathic REM sleep behaviour disorder: a multicentre study. Brain 142, 744–759. doi: 10.1093/brain/awz030, PMID: 30789229 PMC6391615

[ref37] Rechtschaffen A. Gilliland M. A. Bergmann B. M. Winter J. B. (1983). Physiological correlates of prolonged sleep deprivation in rats. Science 221, 182–184. doi: 10.1126/science.6857280, PMID: 6857280

[ref38] Reid K. J. Baron K. G. Lu B. Naylor E. Wolfe L. Zee P. C. (2010). Aerobic exercise improves self-reported sleep and quality of life in older adults with insomnia. Sleep Med. 11, 934–940. doi: 10.1016/j.sleep.2010.04.014, PMID: 20813580 PMC2992829

[ref39] Roth T. (2007). Insomnia: definition, prevalence, etiology, and consequences. J. Clin. Sleep Med. 3, S7–S10. 17824495 PMC1978319

[ref40] Ryden A. M. Martin J. L. Matsuwaka S. Fung C. H. Dzierzewski J. M. Song Y.. (2019). Insomnia disorder among older veterans: results of a postal survey. J. Clin. Sleep Med. 15, 543–551. doi: 10.5664/jcsm.7710, PMID: 30952212 PMC6457506

[ref41] Sathyanarayanan A. Mueller T. T. Ali Moni M. Schueler K. ECNP TWG Network members Baune B. T.. (2023). Multi-omics data integration methods and their applications in psychiatric disorders. Eur. Neuropsychopharmacol. 69, 26–46. doi: 10.1016/j.euroneuro.2023.01.001, PMID: 36706689

[ref42] Sen P. Molinero-Perez A. O'Riordan K. J. McCafferty C. P. O'Halloran K. D. Cryan J. F. (2021). Microbiota and sleep: awakening the gut feeling. Trends Mol. Med. 27, 935–945. doi: 10.1016/j.molmed.2021.07.004, PMID: 34364787

[ref43] Seow L. S. E. Verma S. K. Mok Y. M. Kumar S. Chang S. Satghare P.. (2018). Evaluating DSM-5 insomnia disorder and the treatment of sleep problems in a psychiatric population. J. Clin. Sleep Med. 14, 237–244. doi: 10.5664/jcsm.6942, PMID: 29394962 PMC5786843

[ref44] Smith R. P. Easson C. Lyle S. M. Kapoor R. Donnelly C. P. Davidson E. J.. (2019). Gut microbiome diversity is associated with sleep physiology in humans. PLoS One 14:e0222394. doi: 10.1371/journal.pone.0222394, PMID: 31589627 PMC6779243

[ref45] Soysal P. Smith L. Dokuzlar O. Isik A. T. (2019). Relationship between nutritional status and insomnia severity in older adults. J. Am. Med. Dir. Assoc. 20, 1593–1598. doi: 10.1016/j.jamda.2019.03.030, PMID: 31109907

[ref46] Suh S. Cho N. Zhang J. (2018). Sex differences in insomnia: from epidemiology and Etiology to intervention. Curr. Psychiatry Rep. 20:69. doi: 10.1007/s11920-018-0940-9 30094679

[ref47] Sutton E. L. (2021). Insomnia. Ann. Intern. Med. 174:ITC33–ITC48. doi: 10.7326/AITC202103160 33683929

[ref48] Tian P. Chen Y. Zhu H. Wang L. Qian X. Zou R.. (2022). *Bifidobacterium breve* CCFM1025 attenuates major depression disorder via regulating gut microbiome and tryptophan metabolism: a randomized clinical trial. Brain Behav. Immun. 100, 233–241. doi: 10.1016/j.bbi.2021.11.023 34875345

[ref49] Tkach S. Dorofeyev A. Kuzenko I. Falalyeyeva T. Tsyryuk O. Kovalchuk O.. (2023). Efficacy and safety of fecal microbiota transplantation via colonoscopy as add-on therapy in patients with mild-to-moderate ulcerative colitis: a randomized clinical trial. Front. Med. (Lausanne) 9:1049849. doi: 10.3389/fmed.2022.1049849, PMID: 36714101 PMC9877446

[ref50] van Straten A. van der Zweerde T. Kleiboer A. Cuijpers P. Morin C. M. Lancee J. (2018). Cognitive and behavioral therapies in the treatment of insomnia: a meta-analysis. Sleep Med. Rev. 38, 3–16. doi: 10.1016/j.smrv.2017.02.001 28392168

[ref51] Wang Z. Chen W. H. Li S. X. He Z. M. Zhu W. L. Ji Y. B.. (2021). Gut microbiota modulates the inflammatory response and cognitive impairment induced by sleep deprivation. Mol. Psychiatry 26, 6277–6292. doi: 10.1038/s41380-021-01113-1, PMID: 33963281

[ref52] Wang Y. van de Wouw M. Drogos L. Vaghef-Mehrabani E. Reimer R. A. Tomfohr-Madsen L.. (2022). Sleep and the gut microbiota in preschool-aged children. Sleep 45:zsac020. doi: 10.1093/sleep/zsac020, PMID: 35037059 PMC9189981

[ref53] Weingarden A. González A. Vázquez-Baeza Y. Weiss S. Humphry G. Berg-Lyons D.. (2015). Dynamic changes in short- and long-term bacterial composition following fecal microbiota transplantation for recurrent *Clostridium difficile* infection. Microbiome 3:10. doi: 10.1186/s40168-015-0070-0, PMID: 25825673 PMC4378022

[ref54] Zhang Z. Li Q. Zhang S. Liu Y. Lu G. Wen Q.. (2023). Washed microbiota transplantation targeting both gastrointestinal and extraintestinal symptoms in patients with irritable bowel syndrome. Prog. Neuro-Psychopharmacol. Biol. Psychiatry 127:110839. doi: 10.1016/j.pnpbp.2023.110839, PMID: 37562707

[ref55] Zhang T. Xie X. Li Q. Zhang L. Chen Y. Ji G. J.. (2022). Hypogyrification in generalized anxiety disorder and associated with insomnia symptoms. Nat sci. Sleep 14:1009. Published 2022 May 25. doi: 10.2147/NSS.S358763, PMID: 35642211 PMC9148579

[ref56] Zhang B. Yang L. Ning H. Cao M. Chen Z. Chen Q.. (2023). A matching strategy to guide donor selection for ulcerative colitis in Fecal microbiota transplantation: meta-analysis and analytic hierarchy process. Microbiol. Spectr. 11:e0215921. doi: 10.1128/spectrum.02159-21, PMID: 36472435 PMC9927247

